# Lung cancer risk in relation to indicative radon atlas metrics in Northern Ireland: a population-based case–control study using secondary data

**DOI:** 10.1007/s10653-026-03153-4

**Published:** 2026-04-10

**Authors:** Claire M. Delargy, Helen G. Coleman, Quentin G. Crowley, Damien Bennett, Javier Elio, Deirdre Fitzpatrick, Helen Mitchell, Sara M. Wallace, Rawan A. N. Alhattab, Angela Scott, Bernadette McGuinness, Ruth F. Hunter, Gareth J. McKay, Daniel R. S. Middleton

**Affiliations:** 1 Centre for Public Health, Institute of Clinical Sciences B, Queen’s University Belfast, Royal Victoria Hospital, Belfast, BT12 6BA Northern Ireland; 2 Geology, School of Natural Sciences, Trinity College Dublin, Dublin, Republic of Ireland; 3 Centre for Public Health, Northern Ireland Cancer Registry, Queen’s University Belfast, Belfast, Northern Ireland; 4 Western Norway University of Applied Sciences, Bergen, Norway

**Keywords:** Radon, Lung cancer, Case–control, Mapping

## Abstract

Radon is an established lung carcinogen, but more research is needed to inform whether area-based measures of radon exposure reflect underlying cancer risk. This study, the first of its kind in the UK, aimed to investigate the association between a nationwide indicative radon atlas and lung cancer risk in Northern Ireland, a region in which lung cancer screening programmes are under design. A population-based case–control study was conducted. Primary lung cancer cases (*n* = 1,687) from the Northern Ireland Cancer Registry diagnosed in 2006 and 2014 were compared with 8,094 non-lung cancer controls from a large cohort. Radon exposure data were extracted from the Indicative Atlas of Radon, which categorises 1 km^2^ areas by the percentage of households exceeding 200 Bq/m^3^. Exposure data were linked to participants by postcode. Logistic regression models adjusted for age, sex, smoking status, deprivation and PM_2.5_ exposure were used to estimate odds ratios (ORs) and 95% confidence intervals (CIs) for lung cancer. Individuals residing in high radon exposure areas (Atlas Class 6) had a greater than two-fold lung cancer risk (adjusted OR: 2.24, 95% CI: 1.25, 3.92) compared to residents in all other areas. This association was revealed after adjusting for PM_2.5_. This study supports the utility of an indicative radon atlas in reflecting underlying population lung cancer risk associated with radon, while highlighting constraints inherent to atlas-based exposure classification and secondary data.

**Supplementary Information:**

The online version contains supplementary material available at 10.1007/s10653-026-03153-4.

## Introduction

Lung cancer is the most common cancer and the leading cause of cancer death worldwide, accounting for one in eight cancer diagnoses and almost one in five cancer-related deaths globally in 2022 (Bray et al., 2024). Tobacco smoking remains the leading cause of lung cancer, accounting for an estimated 72% of lung cancer cases in the UK (Brown et al., 2018). However, as smoking prevalence declines in many populations (Department of Health. 2010), investigating other risk factors is important to address the remaining lung cancer burden. While factors such as air pollution are likely substantial contributors to lung cancer incidence in non-smokers (Cufari et al., 2017; Pelosof et al., 2017), the role of other environmental exposures, including radon, requires continued examination.

Radon (predominantly Radon-222) is a naturally occurring radioactive gas produced by the decay of uranium in soil, rock, and water. Radon was evaluated as an IARC Group 1 carcinogen to the lung in 1988 (IARC, 1988). Radon accumulates in unventilated indoor areas, such as basements, entering the body through inhalation, and emitting alpha particles which interact with lung cells, causing DNA damage that can lead to cancer (IARC, 2009). Radon and its radioactive progeny are thought to be responsible for approximately 50% of human natural radiation exposure (National Council on Radiation Protection & Measurements, 2006; World Health Organisation. 2009).

The association between residential radon and lung cancer risk has been investigated in a large, pooled analysis from 13 European case–control studies (Darby et al., 2005). The risk of lung cancer was found to increase by 16% per 100 Bq/m^3^ increment in radon exposure, with no evidence of a lower threshold for lung cancer risk even at relatively lower levels of radon. Only one of the 13 European studies took place in the UK, in an isolated high radon risk area (Darby et al., 1998). These estimates (Darby et al., 2005), and others from North America (Hystad et al., 2014; Krewski et al., 2005) and Asia (Lubin et al., 2004), suggest that between 3–14% of lung cancer cases are attributable to radon exposure, depending on the range of indoor radon concentration values (World Health Organisation. 2009). However, since most of these studies were conducted outside the UK, they may not fully account for other factors that influence local radon concentrations, such as geology, climate and indoor ventilation practices (World Health Organisation. 2009). For example, in Northern Ireland, the population-weighted average radon concentration was reported to be 19 Bq/m^3^ in 2016 (Public Health England. 2016), lower than the global average indoor radon concentration of 39 Bq/m^3^ (World Health Organisation. 2009), however an estimated 1,300 homes were at/above the action level of 200 Bq/m^3^ (Public Health England. 2016).

High-resolution radon maps exist in the UK, including the UKHSA/BGS Indicative Radon Atlas (British Geological Survey, 2020), which provides nationwide coverage based on over 23,000 indoor measurements. This atlas is a valuable tool for identifying broad geographic patterns in indoor radon measurements. However, to our knowledge, it has not previously been integrated with cancer statistics to investigate how well underlying radon-attributable lung cancer risk variation is reflected. Readily available estimates of the geospatial association between lung cancer risk and radon exposure assessment are important for informing targeted public health interventions, environmental planning policies, and lung cancer screening efforts in high-risk areas, especially in the absence of individual-level household radon monitoring data.

The aim of this study was not to establish a causal relationship between radon exposure and lung cancer, which is already well documented, nor to estimate individual-level radon exposure. Rather, this population-based case–control study aimed to evaluate whether readily available, population-level geospatial radon metrics reflect underlying contrasts in lung cancer risk at the population level in Northern Ireland. In doing so, the study assesses the epidemiological utility of such metrics, despite their known limitations, in settings where individual residential radon measurements or long-term residential histories are unavailable.

## Materials and methods

### Study design and data sources

This population-based case–control study was conducted in Northern Ireland, a UK region with a population of 1.9 million (Office for National Statistics, 2024). Lung cancer cases were identified from the population-based Northern Ireland Cancer Registry (NICR) (Northern Ireland Cancer Registry, 2024). Cases were compared with cancer-free controls from the Northern Ireland Cohort for the Longitudinal study of Ageing (NICOLA), which comprises a sample of more than 8500 adults aged over 50 years (Neville et al., 2023).

### Ethical approval

Ethical approval for the Northern Ireland Cohort for the NICOLA study was obtained from the School of Medicine, Dentistry and Biomedical Sciences Research Ethics Committee, Queen’s University Belfast (Reference: 12/23). Written informed consent for the use of data collected via questionnaires and during the clinical based health assessment was obtained from participants. All NICOLA participants were sent a letter regarding the intended use of their health records and were given clear means by which to opt out via a written form. Data were not extracted for participants who opted out of this process or who were not sent the letter regarding data linkage. The NICR has ethical approval from the Office for Research Ethics Committees of Northern Ireland (Reference: 20/NI/0132), for the collection and use of routinely collected data relating to cancer patients within health and social care research. Both NICOLA and the NICR have internal application processes that were approved to facilitate the data linkage for this analytical study.

### Case definition

Incident lung cancer cases were identified using the International Classification of Diseases for Oncology, 3rd Edition (ICD-O-3) codes C34.0–C34.9 from the NICR. Cases diagnosed in 2006 and 2014 were included, as these years correspond to lung cancer audits and therefore had smoking data available (McKee et al., 2014). Patients with carcinoid tumours or for whom death certification was the only information source for diagnosis were excluded.

### Control definition

Controls were selected from the NICOLA cohort. Those recruited during Wave 1 (2013–2015) were selected. The NICOLA cohort sample includes adults aged ≥ 50 years living in private households in Northern Ireland (Neville et al., 2023). Eligible individuals were identified using the Business Services Organisation (BSO) General Practitioner Register Database. The sample was geographically stratified, with addresses ordered within postcode geography, and a systematic fixed-interval sample was drawn. The sample was then merged with the Pointer Database (Land & Property Services. 2025), maintained by Land and Property Services, which provides a standardised address identifier for every property in Northern Ireland. The NICOLA study website contains details of all the data that are available through a fully searchable data dictionary (NICOLA Study, 2024).

### Inclusion/exclusion criteria for cases and controls

**Fig. 1:**
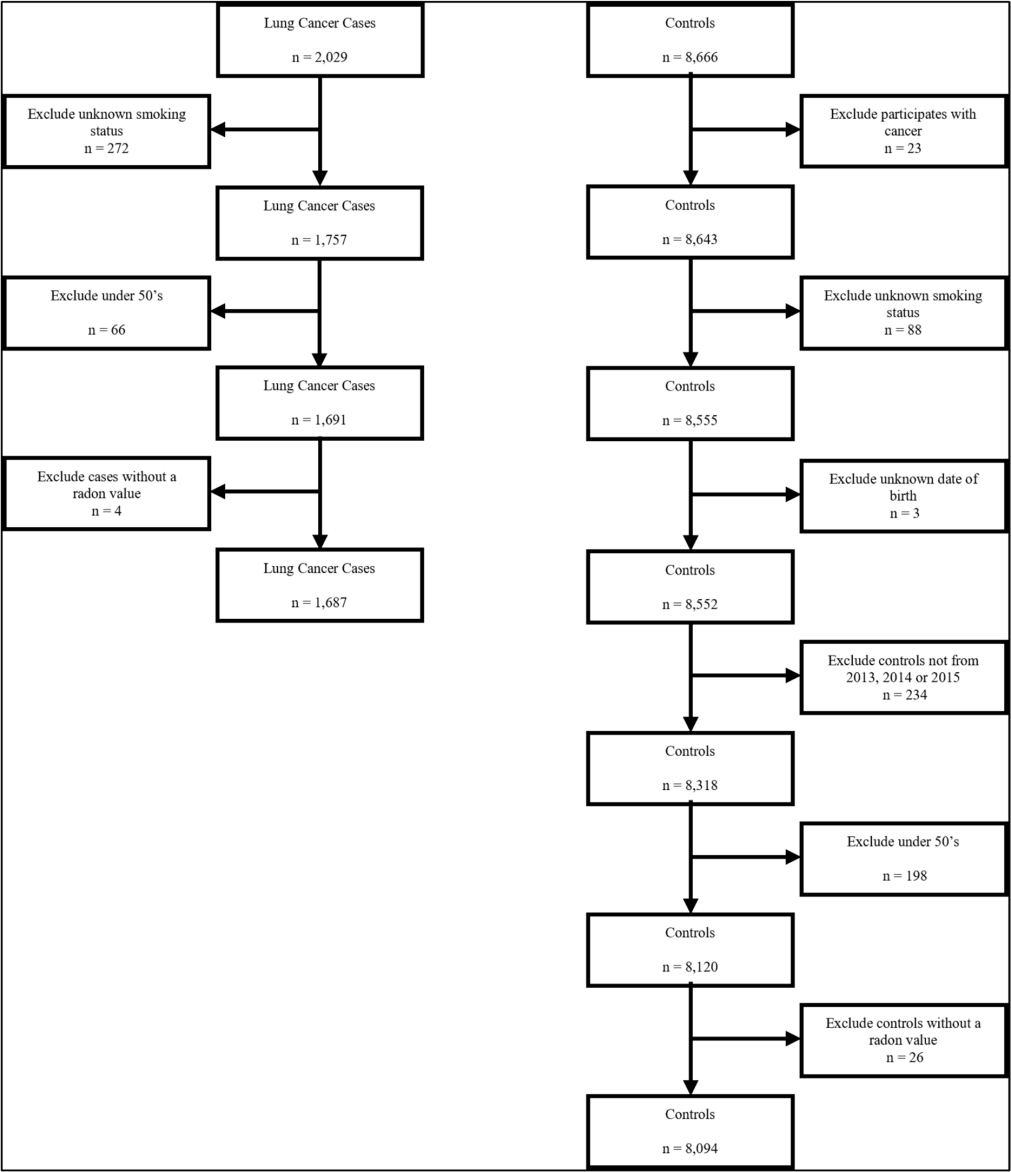
Flowchart illustrating the inclusion and exclusion criteria for lung cancer cases and controls. Cases were identified from the Northern Ireland Cancer Registry (NICR) and controls from the Northern Ireland Cohort for the Longitudinal study of Ageing (NICOLA)

The exclusion criteria for both cases and controls are depicted in the flow diagram shown in Fig. 1. Of 2029 lung cancer cases, 272 were excluded due to missing smoking status. Comparisons with smoking distributions from previous audits indicated that smoking prevalence was within the expected range after these exclusions. An additional 66 cases under the age of 50 were excluded to better align with the control group—i.e. to prevent age brackets with no representation in either cases or controls. Four cases were excluded due to lack of a radon exposure estimate data, resulting in a final sample of 1687 cases.

Figure 1 further illustrates the exclusion of 23 cancer cases from the control group. As with the cases, 88 controls with missing smoking data and 198 individuals under the age of 50 were excluded. A further three controls who declined to report their date of birth were excluded. Additionally, 234 controls who did not complete their NICOLA interview in 2013, 2014, or 2015 were excluded to ensure control data corresponded to a recruitment time-period within ten years of all case diagnoses. A final 26 controls for whom a radon exposure estimate data could not be determined were excluded, resulting in 8094 controls.

### Radon exposure characterisation

Radon exposure in this study was characterised using a geospatial radon atlas, which provides freely available area-based geospatial indicators of indoor radon rather than direct measurements at the individual dwelling level. These indicators are designed to capture broad spatial patterns in radon potential and are subject to uncertainty at fine spatial scales. Accordingly, the atlas-based categories were used here to examine whether population-level exposure contrasts, as defined by this routinely used resource, correspond to underlying differences in lung cancer risk. This results in a partially ecological exposure assessment within an individual-level case–control study.

The Indicative Atlas of Radon, published by the UK Health Security Agency (UKHSA), the Geology Survey of Northern Ireland (GSNI) and British Geological Survey (BGS), were used as the radon exposure data (Appleton et al., 2022). The Northern Ireland dataset is based on measurements from over 23,000 homes in Northern Ireland conducted between late 1999 and early 2009 (Green et al., 2009). Although the atlas measurements long pre-dated some case diagnoses and control recruitment, radon potential maps are generally considered stable over decadal timescales, and this period is still relevant given the long latency of radon-related lung cancer. Each 1 km^2^ area is classed based on the estimated percentage of homes at or above the action level of 200 Bq/m^3^: Class 1 (< 1%), Class 2 (1– < 3%), Class 3 (3– < 5%), Class 4 (5– < 10%), Class 5 (10– < 30%), and Class 6 (30–100%). These categories are fixed within the published atlas and were not available for re-categorisation within the present study.

**Fig. 2:**
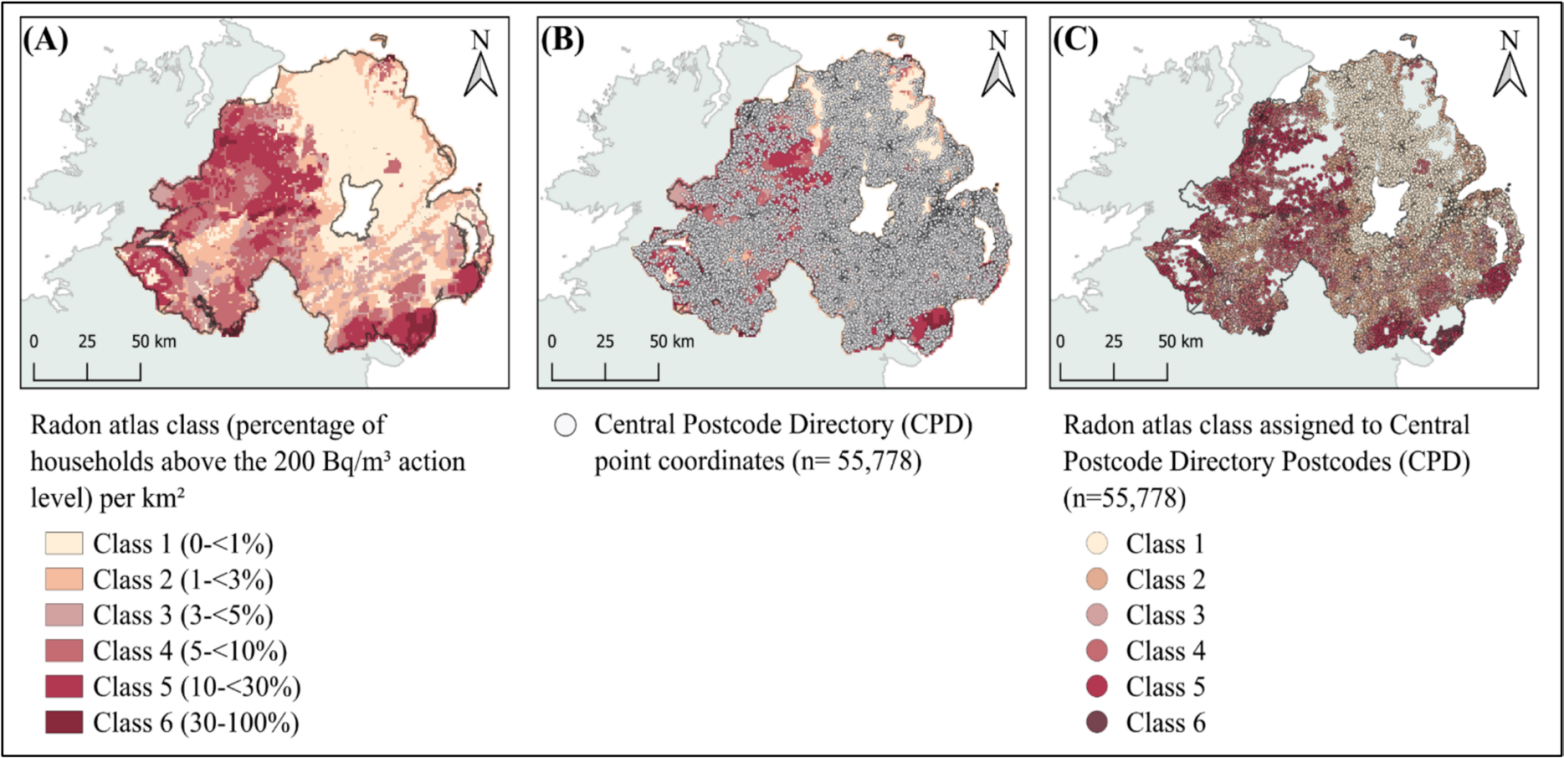
Geospatial radon exposure classification. Map (A) shows radon atlas classification per square kilometre, showing the class which is based on the percentage of homes exceeding 200 Bq/m^3^. Map (B) shows the distribution of Central Postcode Directory (CPD) point coordinates (*n* = 55,778) across Northern Ireland. Map (C) shows radon atlas classification assigned to each postcode. Postcodes of study participants were then extracted from this radon atlas-linked CPD dataset. Contains British Geological Survey materials © UKRI 2025. Radon Potential classification UK Health Security Agency © Crown copyright 2025

The Indicative Atlas of Radon dataset (British Geological Survey 2020), available from the BGS as a shapefile of GIS polygon data at 1 km^2^ resolution, was imported into a Geographic Information System (GIS) using the Quantum GIS (QGIS) software package (version 3.36.1) and converted into raster format for point sampling. To extract radon values, postcode coordinates for the 55,778 active postcodes registered in the Northern Ireland Central Postcode Directory (CPD) (Northern Ireland Statistics & Research Agency. 2024), were imported into QGIS. The ‘Sample Raster Values’ tool within the Point Sampling Tool plugin was then used to obtain the radon class values from their corresponding raster grid cells. This process is illustrated in the maps presented in Fig. 2. The postcodes provided by the NICOLA participants indicating their address at time of interview, or from the NICR at time of diagnosis for lung cancer cases, were then merged with the CPD dataset which included radon class joined to each postcode.

### Covariates

For cancer cases, data on age, sex and postcode at time of diagnosis are collected by the NICR. Smoking status (current/past/never) was obtained as part of the lung cancer audits by reviewing linked medical records (Bannon & Gavin, 2006; McKee et al., 2014). For controls, the same variables were collected during computer-assisted personal interviews (CAPI) conducted in participants’ homes as part of the NICOLA study. The Northern Ireland Multiple Deprivation Measure (NIMDM) 2010 was used as a socio-economic indicator to indicate deprivation quintiles for both cases and controls (Northern Ireland Multiple Deprivation Measure, 2010). This was merged with the CPD and radon dataset, so each postcode had an associated deprivation score.

Annual mean ambient particulate matter with a diameter of 2.5 µm or less (PM_2.5_) concentrations for 2006–2013 were obtained from the Department for Environment, Food and Rural Affairs (DEFRA) (Loader & Willis, 2013; Loader et al., 2006; Mapping & data for local authorities, 2025). These data were linked to participants via postcode in the same manner as the radon atlas (Alhattab et al., 2025).

### Statistical analysis

Descriptive statistics were calculated for both cases and controls. The primary exposure measure was based on binary categories of radon atlas classifications: ‘low exposure’ (Classes 1–5) and ‘high exposure’ (Class 6). This dichotomisation reflects the fixed structure of the atlas and the largest available exposure contrast within this framework. Although Class 6 does not imply uniformly elevated exposure, it captures the greatest concentration of high-radon dwellings and therefore most individuals experiencing the highest residential radon levels, whereas in Class 5 and below elevated radon remains a minority characteristic. Given this heterogeneity, any true association between radon and lung cancer would be expected to attenuate observed exposure contrasts rather than inflate them. To assess the robustness of this dichotomisation, supplementary analyses were conducted using three exposure categories derived from the atlas classification (Classes 1–4, Class 5, and Class 6). This alternative grouping allowed Class 5 to be evaluated separately from the highest exposure category while maintaining sufficient numbers for stable estimation (Supplementary Table 1).

Chi-squared tests were used to investigate associations between potential confounders and the radon exposure categories in controls. Odds ratios (ORs) and their 95% confidence intervals (CI) of lung cancer risk associated with radon exposure were then estimated using logistic regression models adjusting for age (5-year age bands), sex (binary), smoking status (current/past/never), deprivation as a proxy for socio-economic status (Quintiles 1 to 5) and PM_2.5_ exposure (Quintiles 1 to 5). The air pollution groupings were calculated based on PM_2.5_ quintiles of the control groups (1: < 6.87 µg/m3, 2: 6.87—< 7.73 µg/m3, 3: 7.73—< 8.94 µg/m3, 4: 8.94—< 10.75 µg/m3, 5: 10.75 + µg/m3). This variable was included as a covariate in the analysis to adjust for potential confounding by ambient air pollution.

To control for the difference in sex and age between cases and controls, a matched design was also undertaken, pairing each case with up to four controls of the same sex and within ± one year of age. The previously described models were repeated, again using conditional logistic regression, shown in Supplementary Fig. 1.

Subgroup analyses were conducted separately for lung cancer cases diagnosed in 2006 and 2014 to examine the association of radon exposure with lung cancer risk within each year-specific series of cases. This stratification was undertaken to assess the consistency of the radon exposure and lung cancer risk association across these two time periods in Northern Ireland. Furthermore, sensitivity analyses were conducted to account for the partial lack of calendar overlap between case diagnosis and control recruitment years by restricting analyses to participants from 2014, the only year in which both cases and controls were available.

Further subgroup analyses were conducted to examine whether the relationship between radon exposure and lung cancer risk differs by demographics including age, sex, smoking status, histological subtype of lung cancer, and Health and Social Care Trusts (HSCT) which had participants in both high and low radon exposure categories (Northern, Southern, and Western HSCT).

As an additional exercise, the Population Attributable Fraction (PAF, %) was estimated using a standard model-based approach using fully adjusted ORs. In the present context, given the limitations of the exposure dataset, PAFs should be interpreted as illustrative rather than as precise estimates of preventable disease burden. Miettinen’s formula (Miettinen, 1974) shown in Eq. 1, was used to estimate the proportion of lung cancer cases attributable to residing in radon atlas Class 6 using a binary classification, comparing Classes 1–5 to Class 6:

1 $${\mathrm{PAF}} = {\mathrm{pc}} \, \times ({\mathrm{OR}}_{\mathrm{adj}} -1)/ {\mathrm{OR}}_{\mathrm{adj}}$$

where PAF is the population attributable fraction, pc is the proportion of cases that reside in Class 6, and OR_adj_ is the fully adjusted OR. The PAF estimates were reported based on the point estimate of the odds ratio (OR) and the lower and upper bounds of the 95% confidence interval (95% CI). This yields an estimate of the percentage of avoidable cases if Class 6 residents lived in other classes.

All statistics were performed in the R programming language (version 4.2.2) with the R Studio interface (version 2023.12.1).

## Results

### Participant and exposure characteristics

A total of 1687 lung cancer cases and 8094 controls were included in the study, with key demographic and exposure variables summarised in Table 1. Cases, all diagnosed in 2006 or 2014, had a higher proportion of males (58%). Controls had a higher proportion of females (55%) and were primarily recruited in 2015. Lung cancer cases were generally older than controls (71 ± 9 Vs. 65 ± 10 years). Cases had a higher proportion of ever smokers (current or past); 94% in comparison to 52% of controls. Lung cancer cases were twice as likely to reside in more deprived areas, with a greater proportion in Quintile 5 (most deprived) compared to controls (30% Vs. 15%). For both cases and controls, 99% were classified as having low radon exposure, with only 1% falling into the high exposure category.

**Table 1:** Demographic and exposure characteristics of cases and controls

Category	Cases *n*= 1,687	Controls *n*= 8,094		*P*-values*
*Sex*				
Male	978 (58%)	3663 (45%)		
Female	709 (42%)	4431 (55%)		
				< 0.001
Recruitment year**				
2006	769 (46%)	0 (0%)		
2013	0 (0%)	32 (0.4%)		
2014	918 (54%)	3736 (46%)		
2015	0 (0%)	4326 (53%)		
				< 0.001
*Age*				
Mean age (± SD)	71 (± 9)	65 (± 10)		
Range	50—96	50—99		
				< 0.001
*Smoking status*				
Current	861 (51%)	1351 (17%)		
Past	730 (43%)	2824 (35%)		
Never	96 (6%)	3919 (48%)		
				< 0.001
*10-Year age band*				
50–59	199 (12%)	2748 (34%)		
60–69	509 (30%)	2735 (34%)		
70–79	696 (41%)	1794 (22%)		
80–89	269 (16%)	728 (9%)		
≥ 90	14 (0.8%)	89 (1%)		
				< 0.001
*Deprivation quintiles*				
Quintile 1 (Least)	208 (12%)	1772 (22%)		
Quintile 2	299 (18%)	1750 (22%)		
Quintile 3	308 (18%)	1806 (22%)		
Quintile 4	367 (22%)	1575 (19%)		
Quintile 5 (Most)	505 (30%)	1191 (15%)		
				< 0.001
*Radon exposure*				
Low	1666 (99%)	8024 (99%)		
High	21 (1%)	70 (0.9%)		
			0.139	

^*^For chi-squared or t-tests comparing cases with controls

^**^For cancer cases, the recruitment year refers to the year of diagnosis from the Northern Ireland Cancer Registry (NICR)

To explore potential confounding variables for subsequent adjustment in regression models, key demographic and exposure variables were cross tabulated with radon exposure categories and investigated using Chi-squared tests as shown in Table 2. Significant associations were observed for deprivation quintile (*p* < 0.001) and air pollution quintile (*p* < 0.001), but no significant associations were found for sex (*p* = 0.60), age band (*p* = 0.45), or smoking status (*p* = 0.26). All these variables were considered in the adjusted regression models to account for potential confounding effects on the relationship between radon exposure and lung cancer risk. Notably, PM_2.5_ exposure was an important negative confounder likely due to its geospatial pattern indicating that for Northern Ireland, urban areas with high air pollution tend to have lower radon exposure.

**Table 2:** Radon exposure in controls, overall and by potential confounding factors

Category	Radon exposure		*P*-values
	low	High	
	*n* = 8,024	*n* = 70	
*Sex*			
Male	3634 (99.2%)	29 (0.8%)	
Female	4390 (99.1%)	41 (0.9%)	
			0.60
*10-Year age band*			
50–59	2729 (99.3%)	19 (0.7%)	
60–69	2711 (99.1%)	24 (0.9%)	
70–79	1777 (99.1%)	17 (0.9%)	
80–89	718 (98.6%)	10 (1.4%)	
≥ 90	89 (100%)	0 (0%)	
			0.45
*Smoking status*			
Current	1344 (99.5%)	7 (0.5%)	
Past	2800 (99.2%)	24 (0.8%)	
Never	3880 (99%)	39 (1%)	
			0.26
*Deprivation quintiles*			
Quintile 1 (Least)	1772 (100%)	0 (0%)	
Quintile 2	1746 (99.8%)	4 (0.2%)	
Quintile 3	1784 (98.8%)	22 (1.2%)	
Quintile 4	1536 (97.5%)	39 (2.5%)	
Quintile 5 (Most)	1186 (99.6%)	5 (0.4%)	
			< 0.001
*Air pollution quintiles*			
Quintile 1 (Least)	1592 (98.3%)	28 (1.7%)	
Quintile 2	1602 (98.8%)	20 (1.2%)	
Quintile 3	1592 (98.7%)	21 (1.3%)	
Quintile 4	1623 (100%)	0 (0%)	
Quintile 5 (Most)	1609 (100%)	0 (0%)	
			< 0.001

### Radon atlas metrics and LC risk

Odds ratios (ORs) and corresponding 95% confidence intervals (95% CI) of lung cancer associated with radon exposure are presented in Table 3. Crude ORs (Model 1) showed a non-significant increase in lung cancer risk for high radon exposure compared to low radon exposure (OR: 1.44; 95% CI: 0.86, 2.32). After adjusting for sex and age (Model 2), the ORs were slightly attenuated (OR: 1.37; 95% CI: 0.80, 2.24). In the fully adjusted model (Model 3), which adjusted for sex, age, smoking status, air pollution quintile and deprivation quintile, the OR increased to 2.24 (95% CI: 1.25, 3.92), showing a significant excess risk of lung cancer associated with high radon exposure. When examining each recruitment year separately for cases (2006 and 2014), similar trends were found for the fully adjusted models, shown in Table 3. The 2006 subgroup demonstrated a stronger and statistically significant association (OR: 2.84; 95% CI: 1.33, 5.61), compared to 2014 (OR: 1.78; 95% CI: 0.81, 3.57). These analyses were conducted using the same control group across both years, with only the case group being restricted by year. When analyses were restricted to participants from 2014, the calendar year in which both cases and controls were available, the association between residence in Radon Atlas Class 6 and lung cancer remained elevated and of similar magnitude, although with wider confidence intervals reflecting the smaller sample size (fully adjusted OR: 2.13; 95% CI: 0.89–4.89) (Table 3).

**Table 3:** Odds ratios (OR) and 95% confidence intervals (CI) for the association of radon exposure with lung cancer risk in Northern Ireland

Category	Cases/	Model 1—crude	Model 2—minimal adjustment*	Model 3—full adjustment**
	Controls (*n*)	OR (95%)	OR (95%)	OR (95%)
*All participants*				
Radon exposure				
Low (Class 1–5)	1666/8024	1 (ref)	1 (ref)	1 (ref)
High (Class 6)	21/ 70	1.44 (0.86—2.32)	1.37 (0.80—2.24)	2.24 (1.25—3.92)
*2014 cases and all controls*				
Radon exposure				
Low (Class 1–5)	908 / 8024	1 (ref)	1 (ref)	1 (ref)
High (Class 6)	Oct-70	1.26 (0.61—2.34)	1.12 (0.53—2.12)	1.78 (0.81—3.57)
*2006 cases and all controls*				
Radon exposure				
Low (Class 1–5)	758 / 8024	1 (ref)	1 (ref)	1 (ref)
High (Class 6)	Nov-70	1.66 (0.83—3.02)	1.65 (0.81—3.06)	2.84 (1.33—5.61)
*Sensitivity analysis—restricted to 2014 cases and 2014 controls*				
Radon exposure				
Low (Class 1–5)	908/3709	1 (ref)	1 (ref)	1 (ref)
High (Class 6)	Oct-27	1.51(0.69—3.03)	1.40 (0.62—2.91)	2.13 (0.89—4.89)

1 case and 7 controls are missing air pollution data and so were excluded from Model 3. (ref) = Reference category

^*^Adjusted for age and sex. ^**^Adjusted for age, sex, smoking status, deprivation and air pollution exposure

Using an alternative exposure categorisation (Classes 1–4, Class 5, and Class 6), no increased odds of lung cancer were observed for Class 5, whereas the association remained elevated for Class 6 (fully adjusted OR: 2.23; 95% CI: 1.24–3.90) (Supplementary Table 1).

### Stratified analyses

**Fig. 3:**
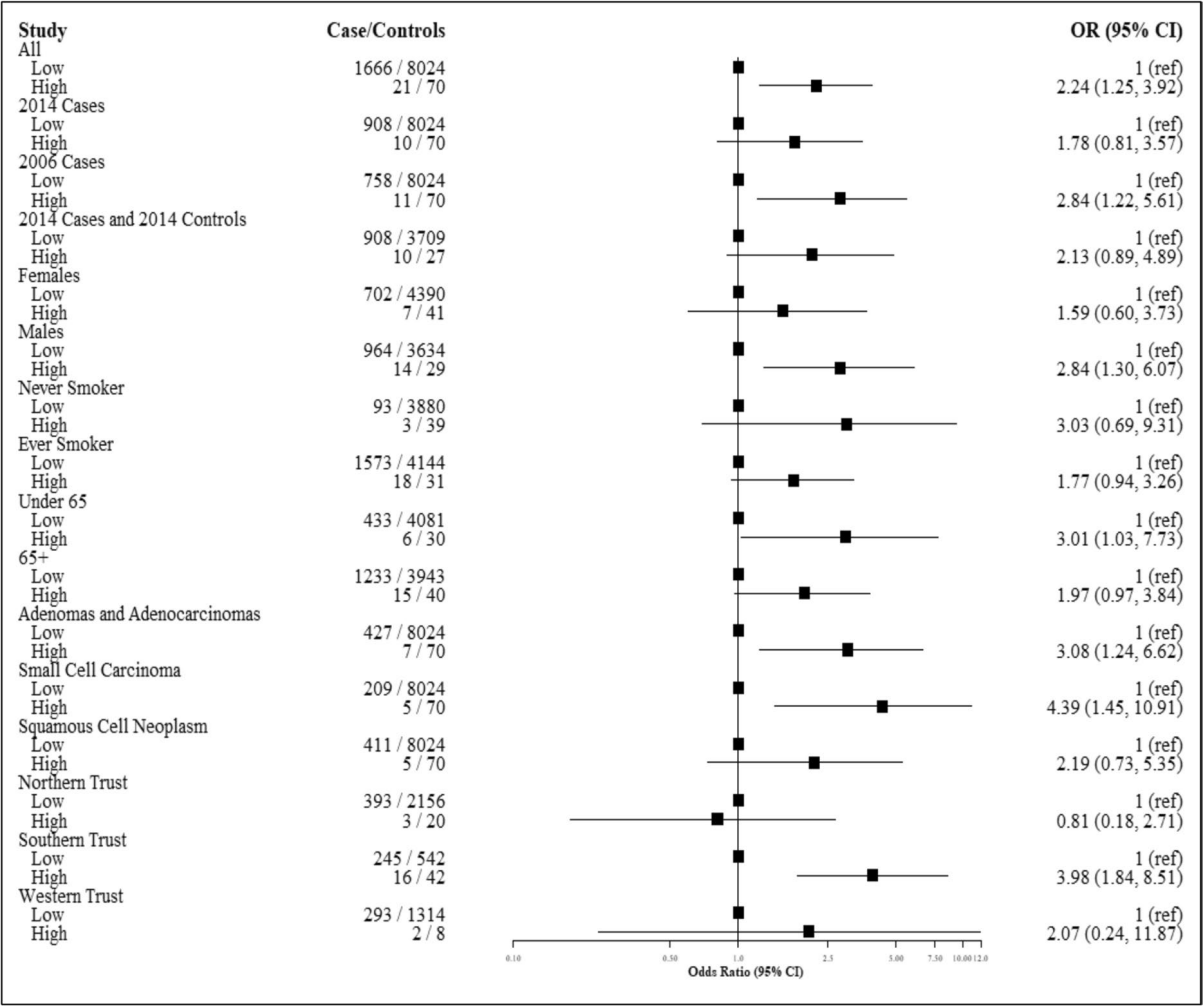
Forest plot of fully adjusted odds ratios (ORs) and 95% confidence intervals (CIs) for the association between radon class and lung cancer. Analyses stratified by recruitment year, sex, smoking status, age group, histological subtype, and Health and Social Care Trust (HSCT). Radon exposure: Low—Classes 1–5 (reference group); High—Class 6

Results of further analyses stratified by recruitment year of cases, sex, smoking status (ever/never), age group, histological subtype and HSCT are shown in Fig. 3. The overall association between high radon exposure and lung cancer risk remained elevated in most subgroups, although statistical significance varied due to smaller sample sizes in these sub-analyses. A stronger, significant effect was observed in males (OR: 2.84; 95% CI: 1.30, 6.07). The strongest association was observed for small cell carcinoma (OR: 4.39, 95% CI: 1.45–10.91), whereas adenocarcinomas (OR: 3.08, 95% CI: 1.24–6.62) and squamous cell carcinomas (OR: 2.19, 95% CI: 0.73–5.35) also showed elevated ORs. The odds ratios varied across different HSCT regions, with the highest observed in the Southern HSCT (OR: 3.98, 95% CI: 1.84–8.51), whereas other trusts showed weaker or non-significant associations.

### Population attributable fraction (PAF)

Population attributable fraction (PAF*)* of lung cancer associated with residing in areas categorised as Radon Atlas Class 6 (30–100% homes above 200 Bq/m^3^) in Northern Ireland was calculated using the fully adjusted OR (Model 3) presented in Table 3 (OR: 2.24; 95% CI: 1.25, 3.92). The percentage of lung cancer cases which could have been theoretically prevented if no individuals resided in Class 6 areas was estimated at 0.7% overall, with a range of 0.2% to 0.9%. Based on the average number of lung cancer cases diagnosed annually in Northern Ireland between 2018 and 2022 (1360 cases) (Northern Ireland Cancer Registry, 2024), this equates to approximately 10 preventable cases annually (range: 3–12) which could potentially have been avoided through radon mitigation efforts in these high-risk areas.

## Discussion

This is the first study to link the UK Indicative Radon Atlas with cancer registry data, and the first case–control study to investigate the association between residential radon and lung cancer risk in Northern Ireland. The analysis showed that residing in high radon areas (Radon Atlas Class 6) was associated with a more than twofold risk of lung cancer (OR: 2.24, 95% CI: 1.25, 3.92), compared to living in Classes 1–5. Additionally, these findings were used to estimate the proportion of lung cancer cases in Northern Ireland attributable to residential radon exposure in radon atlas Class 6 (30–100% of homes exceeding 200 Bq/m^3^). Given the use of area-based exposure indicators and the assumptions underlying radon risk models, these Population Attributable Fraction (PAF) estimates should be regarded as tentative and illustrative of potential population-level impact rather than definitive measures of preventable lung cancer. The PAF was estimated at 0.7% (0.2%–0.9%) overall, but only accounts for those in the highest mapped radon class, with a relatively low proportion of the Northern Irish populace in these areas compared to other investigated settings.

This study offers insights into the relationship between residential radon and lung cancer risk in Northern Ireland. A strength of this research was the use of high-resolution 1 km^2^ gridded radon maps as the primary radon exposure variable. Additionally, the study was able to adjust for key confounders, including age, sex, smoking status, deprivation and, crucially, exposure to air pollution. The use of lung cancer cases from the NICR provided a high-quality, population-based dataset with verified diagnoses, further strengthening the reliability of the study. This research was conducted using open-source geospatial tools, such as QGIS, and the publicly available radon atlas map, meaning it is plausible to consider this radon exposure methodology in large scale population studies.

Several limitations of this study reflect known and unavoidable constraints associated with the use of routinely collected, population-level radon atlas data and were recognised at the design stage; they are reiterated here to aid interpretation of the findings rather than to qualify their validity. The first limitation is the lack of household-level radon exposure data. Indoor radon concentrations can vary substantially between homes within the same area, so use of atlas-based categories is likely to have introduced non-differential exposure misclassification that would tend to attenuate associations. Individual-level data would have provided a more accurate estimate of exposure and offered a clearer understanding of the dose–response relationship between radon and lung cancer in Northern Ireland, however the readily available area-based measure may allow for efficient targeting of populations ‘at-risk’ for prevention and early diagnosis or detection campaigns. The class categorisation of the radon atlas presents a further limitation. The high exposure category (Class 6) included up to 70% of households which are below the action level, which may result in a diluted “exposed” group. This could lead to an underestimation of radon-related lung cancer risk. Only residential postcodes at the time of diagnosis for lung cancer cases were available for this study, limiting the ability to assess lifetime exposure if individuals had moved residence. This is important because radon-related lung cancer risk is more likely to reflect cumulative exposure over many years than residence at a single time point. However, we note that, compared to other regions of the UK, Northern Ireland has a low rate of internal migration—below 2% annually for distances exceeding 10 km (Shuttleworth et al., 2021). Furthermore, the absence of information on occupational exposure to radon could not be accounted for. The regulatory context in Northern Ireland is another important consideration. The Building Regulations 2012 stipulate that radon protection measures must be implemented in radon-affected areas (The Department of Finance. 2012), while the Ionising Radiations Regulations 2017 require action when workplace radon levels exceed 300 Bq/m^3^ in this region (The Department for the Economy, 2017). Additionally, in the Republic of Ireland it has been mandatory since 1998 for any building constructed in a high radon area to include radon barriers (Department of Housing LG & H. 1997). It is possible that individuals in Northern Ireland, particularly those living near the border with the Republic, may have been influenced by these regulations. This could have led to some to proactively implement mitigation measures, such as improving ventilation or installing radon sumps, even in the absence of formal requirements in Northern Ireland. As a result, more recently built individual homes located in higher radon class areas, having already implemented mitigation measures, may record lower than expected radon levels. This could have led to an underestimation of the association between high radon exposure and lung cancer risk in this study. This hypothesis is further supported by the ORs calculated separately for 2006 and 2014 lung cancer cases, seen in Table 3. The higher OR observed in 2006 suggests a stronger association prior to the widespread implementation of radon mitigation measures. Additionally, declining smoking rates over time may also contribute to the lower observed effect in more recent years, due to the synergistic relationship between smoking and radon exposure (Park et al., 2020).

A meta-analysis which included 28 different studies on residential radon exposure and the risk of lung cancer histological subtypes found a similar trend to this study (Li et al., 2020). The meta-analysis reported an overall OR of 1.48 (95% CI: 1.26, 1.73), with small-cell carcinoma showing the highest OR, followed by adenocarcinoma and squamous cell carcinoma, with ORs of 2.03, 1.58, and 1.43, respectively (Li et al., 2020). These findings align with our study, which similarly identified a higher risk of lung cancer in individuals exposed to high radon levels and suggest that the relationship between radon exposure and lung cancer may vary by histological subtype, with small-cell carcinoma being particularly sensitive to radon exposure.

The only other UK-based case–control study investigating residential radon exposure and lung cancer risk was conducted by Darby et al. in southwest England (Darby et al., 1998). They reported an OR of 1.24 (CI: 0.77, 2.01) for radon concentrations of 200–399 Bq/m^3^. However, the smaller sample size for this category (34 cases, 87 controls) and lack of adjustment for air pollution in their analysis likely contributed to the lower, non-statistically significant result. The findings from our study align with international studies. For example, studies from Europe, North America and China have shown increased ORs in higher radon exposure categories, these are shown for reference in Supplementary Fig. 2 (Darby et al., 1998; Krewski et al., 2006; Lorenzo-Gonzalez et al., 2020; Torres-Durán et al., 2014; Wang et al., 2002).

Beyond the methodological implications of our findings, they emphasise the need for continued environmental and public health measures and policies to mitigate radon exposure, particularly in high-risk areas. The public health impact of radon is substantial, with an estimated 1100 deaths annually in the UK attributed to radon alone (Gray et al., 2009). Efforts to reduce radon exposure, alongside comprehensive smoking cessation programs, may be an effective strategy to lower the overall burden of lung cancer in Northern Ireland. Additionally, increasing radon awareness among the public may encourage more households in high-risk areas to act, such as testing their homes for radon. This could contribute to an enhanced accuracy of exposure data. By addressing these gaps, future studies can provide a more accurate understanding of the relationship between radon exposure and lung cancer in Northern Ireland, ultimately leading to better-targeted public health interventions. In England, targeted lung cancer screening is intended to be rolled out by 2029 to adults aged 55–74 years who are current or former smokers (NHS, 2023). Future studies may wish to consider the cost-effectiveness of potentially targeting high-radon exposure areas for public health campaigns, including lung cancer screening (Gray et al., 2009).

In conclusion, this study provides evidence of an association between a readily available resource of mapped residential radon exposure and lung cancer risk in Northern Ireland. Given the increased lung cancer risk in high radon areas, radon atlas metrics may aid identification of populations in whom lung cancer screening programmes, or lung cancer awareness/early diagnosis campaigns could be targeted. The findings of this study also highlight the need for continued efforts to mitigate radon exposure in high-risk areas. Overall, these findings, while not greatly strengthening the causal evidence base for radon and lung cancer, suggest that widely used geospatial radon atlas metrics may capture meaningful population-level risk contrasts, albeit imperfectly, in the absence of individual exposure data.

## Supplementary Information

Below is the link to the electronic supplementary material.

Supplementary file1 (DOCX 108 KB)

## Data Availability

This secondary data analysis made use of existing data resources, including the Northern Ireland Cancer Registry, The NICOLA study, and the UK Indicative Radon Atlas. For access instructions, please contact the authors for advice.
