# Supplementary material for: Lung cancer risk in relation to indicative radon atlas metrics in Northern Ireland: a population-based case–control study using secondary data
Source: Environ Geochem Health. 2026 Apr 10;48(7):295. doi: 10.1007/s10653-026-03153-4 (PMC13068683; doi:10.1007/s10653-026-03153-4)
Supplement: Supplementary file 1 — Supplementary file1 (DOCX 108 KB) [file 10653_2026_3153_MOESM1_ESM.docx]

**Lung cancer risk in relation to indicative radon atlas metrics in Northern Ireland: a population-based case-control study using secondary data**

Claire M. Delargy^1^, Helen G. Coleman^1^, Quentin G. Crowley^2^, Damien Bennett^3^, Javier Elio^4^, Deirdre Fitzpatrick^3^, Helen Mitchell^3^, Sara M. Wallace^1^, Rawan Alhattab^1^, Angela Scott^1^, Bernadette McGuinness^1^, Ruth F. Hunter^1^, Gareth J. McKay^1^, and Daniel R.S. Middleton^1^

^1^Centre for Public Health, Queen’s University Belfast, Belfast, Northern Ireland.

^2^Geology, School of Natural Sciences, Trinity College Dublin, Republic of Ireland.

^3^Northern Ireland Cancer Registry, Centre for Public Health, Queen’s University Belfast, Northern Ireland.
^4^Western Norway University of Applied Sciences, Bergen, Norway.

**Supplementary Information**


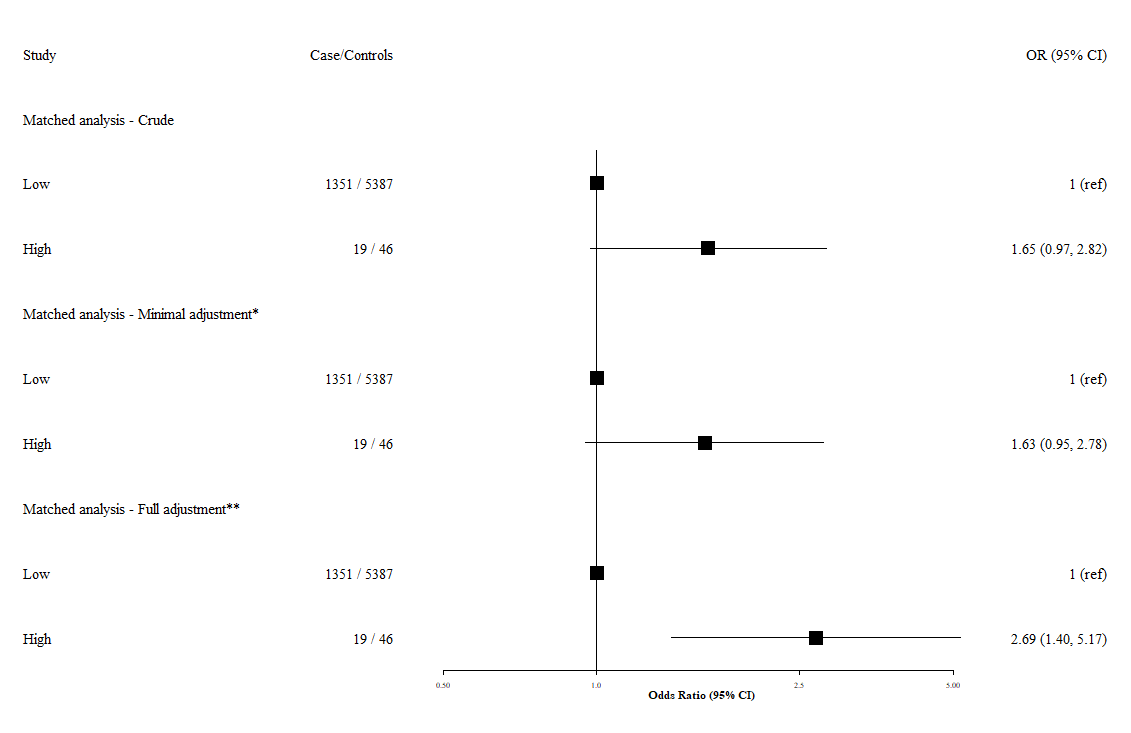

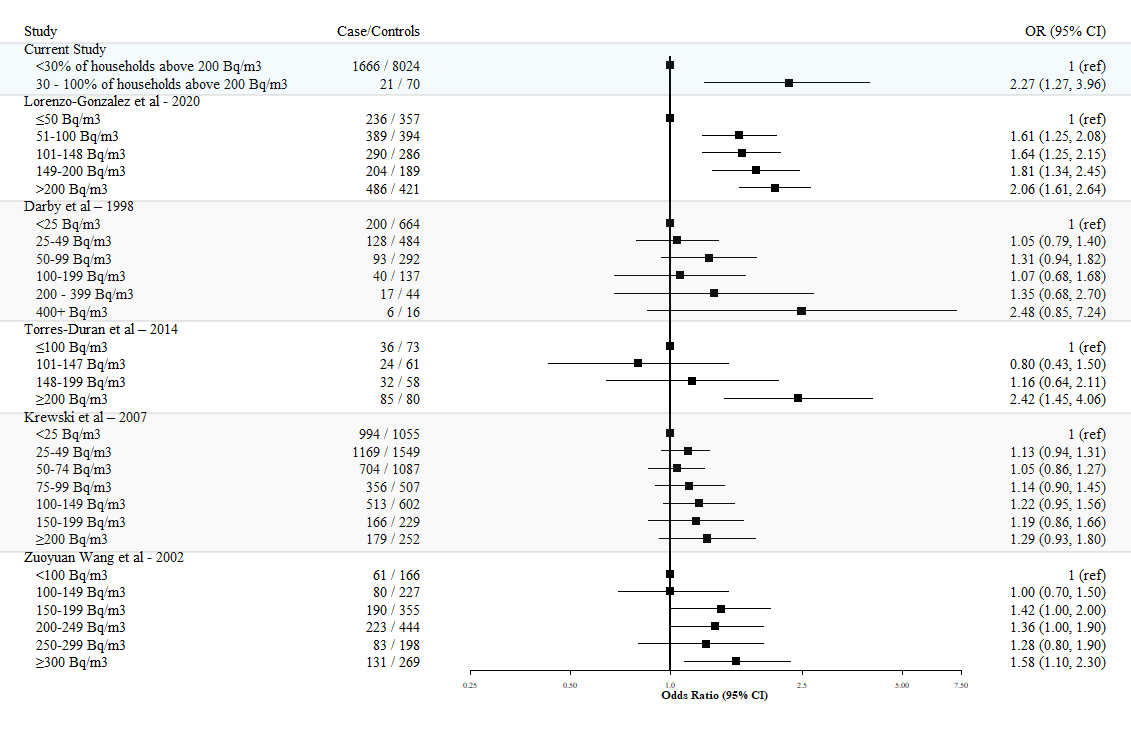


**Supplementary Figure 1.** Odds ratios (OR) and 95% confidence intervals (CI) for the association of radon exposure with lung cancer risk in Northern Ireland using an individually matched case-control design (1:4 matching by sex and age). Results are presented for crude, minimally adjusted, and fully adjusted models. *Odds ratios adjusted for age and sex. **Odds ratios adjusted for age, sex, smoking status, deprivation and air pollution exposure.

**Supplementary Figure 2.** Forest plot showing fully adjusted Odds Ratios (ORs) and 95% Confidence Intervals (CIs) for previous studies investigating residential radon exposure and lung cancer risk.

**Supplementary Table 1.** Odds ratios (OR) and 95% confidence intervals (CI) for the association of radon exposure with lung cancer risk in Northern Ireland.

| Category | Cases/  Controls (*n*) | Model 1 – Crude  OR (95%) | Model 2 – Minimal adjustment*  OR (95%) | Model 3 – Full adjustment**  OR (95%) |
| --- | --- | --- | --- | --- |
| All participants | | | | |
| Radon Exposure  Low (Class 1-4)  Medium (Class 5)  High (Class 6) | 1521 / 7330  145 / 694  21/ 70 | 1 (ref)  1.01 (0.83 – 1.21)  1.45 (0.83 – 1.21) | 1 (ref)  1.03 (0.85 – 1.25)  1.37 (0.81 – 2.25) | 1 (ref)  0.97 (0.78 – 1.21)  2.23 (1.24 – 3.90) |
| 2014 cases and all controls | | | | |
| Radon Exposure  Low (Class 1-4)  Medium (Class 5)  High (Class 6) | 816 / 7330  92 / 694  10 / 70 | 1 (ref)  1.19 (0.94 – 1.49)  1.28 (0.62 – 2.38) | 1 (ref)  1.23 (0.97 – 1.55)  1.14 (0.54 – 2.16) | 1 (ref)  1.10 (0.84 – 1.43)  1.81 (0.82 – 3.63) |
| 2006 cases and all controls | | | | |
| Radon Exposure  Low (Class 1-4)  Medium (Class 5)  High (Class 6) | 705 / 7330  53 / 694  11 / 70 | 1 (ref)  0.79 (0.59 – 1.05)  1.63 (0.82 – 2.97) | 1 (ref)  0.82 (0.60 – 1.08)  1.62 (0.80 – 3.00) | 1 (ref)   - 1. (0.56 – 1.07)   2.75 (1.29 – 5.43) |
| Sensitivity analysis - 2014 cases and 2014 controls | | | | |
| Radon Exposure  Low (Class 1-4)  Medium (Class 5)  High (Class 6) | 816 / 3365  92 / 344  10 / 27 | 1 (ref)  1.10 (0.86 – 1.40)  1.53 (0.70 – 3.07) | 1 (ref)  1.15 (0.89 – 1.48)  1.41 (0.63 – 2.95) | 1 (ref)  1.05 (0.78 – 1.40)  2.15 (0.89 – 4.94) |

*Adjusted for age and sex. **Adjusted for age, sex, smoking status, deprivation and air pollution exposure. 1 case and 7 controls are missing air pollution data and so were excluded from Model 3. (ref) = Reference category.
